# Brain expression of the vascular endothelial growth factor gene family in cognitive aging and alzheimer’s disease

**DOI:** 10.1038/s41380-019-0458-5

**Published:** 2019-07-22

**Authors:** Emily R. Mahoney, Logan Dumitrescu, Annah M. Moore, Francis E. Cambronero, Philip L. De Jager, Mary Ellen I. Koran, Vladislav A. Petyuk, Renã A. S. Robinson, Sandeep Goyal, Julie A. Schneider, David A. Bennett, Angela L. Jefferson, Timothy J. Hohman

**Affiliations:** 1 Vanderbilt Memory and Alzheimer’s Center, Vanderbilt University Medical Center, Nashville, TN USA; 2 Vanderbilt Genetics Institute, Vanderbilt University Medical Center, Nashville, TN USA; 3 Center for Translational & Computational Neuroimmunology, Department of Neurology, Columbia University Medical Center, New York, NY USA; 4 Cell Circuits Program, Broad Institute, Cambridge, MA USA; 5 Stanford Hospital, Department of Radiology, Stanford, CA USA; 6 Biological Sciences Division, Pacific Northwest National Laboratory, Richland, WA USA; 7 Department of Chemistry, Vanderbilt University, Nashville, TN USA; 8 Rush Alzheimer’s Disease Center, Rush University Medical Center, Chicago, IL USA

**Keywords:** Genetics, Neuroscience, Prognostic markers, Genetics, Neuroscience

## Abstract

Vascular endothelial growth factor (VEGF) is associated with the clinical manifestation of Alzheimer’s disease (AD). However, the role of the VEGF gene family in neuroprotection is complex due to the number of biological pathways they regulate. This study explored associations between brain expression of *VEGF* genes with cognitive performance and AD pathology. Genetic, cognitive, and neuropathology data were acquired from the Religious Orders Study and Rush Memory and Aging Project. Expression of ten *VEGF* ligand and receptor genes was quantified using RNA sequencing of prefrontal cortex tissue. Global cognitive composite scores were calculated from 17 neuropsychological tests. β-amyloid and tau burden were measured at autopsy. Participants (*n* = 531) included individuals with normal cognition (*n* = 180), mild cognitive impairment (*n* = 148), or AD dementia (*n* = 203). Mean age at death was 89 years and 37% were male. Higher prefrontal cortex expression of *VEGFB*, *FLT4*, *FLT1*, and *PGF* was associated with worse cognitive trajectories (*p* ≤ 0.01). Increased expression of *VEGFB* and *FLT4* was also associated with lower cognition scores at the last visit before death (*p* ≤ 0.01). *VEGFB*, *FLT4*, and *FLT1* were upregulated among AD dementia compared with normal cognition participants (*p* ≤ 0.03). All four genes associated with cognition related to elevated β-amyloid (*p* ≤ 0.01) and/or tau burden (*p* ≤ 0.03). VEGF ligand and receptor genes, specifically genes relevant to FLT4 and FLT1 receptor signaling, are associated with cognition, longitudinal cognitive decline, and AD neuropathology. Future work should confirm these observations at the protein level to better understand how changes in *VEGF* transcription and translation relate to neurodegenerative disease.

## Introduction

Vascular endothelial growth factors (VEGFs) are important signaling proteins involved in the growth and maintenance of both vascular and neural cells [1]. VEGFA, the founding and most studied member of the VEGF family, appears to protect against cognitive impairment [2], particularly in the context of Alzheimer’s disease (AD) pathology [2–4]. However, the role of VEGFA in neurodegenerative disease is quite complex. In the context of AD dementia, there is mixed evidence in the literature for both up- and downregulation of VEGFA gene and protein expression in the brain, blood, and cerebrospinal fluid (CSF). For example, protein levels of VEGFA have been reported to be higher among AD dementia cases compared with controls in CSF [5], plasma [6], and the medial parietal cortex [7]. Moreover, higher VEGF levels in the AD brain have been associated with loss of pericytes [8], increased blood–brain barrier permeability [8], and more severe tangle pathology [9].

Yet, there is also evidence of downregulation of VEGFA in the context of AD pathology, depending on the brain region sampled and the isoform of VEGFA. For example, one study observed higher VEGF_189_ levels and lower VEGF_121_ levels in the hippocampus of AD cases compared with controls [10]. Beyond tissue expression levels in the brain, there is also evidence of fewer VEGFA-positive capillaries in AD brains compared with controls [11], and an association between lower levels of ventricular fluid VEGF protein levels and a higher tangle burden at autopsy [12]. Additional evidence outside of the brain also suggests that VEGFA levels may be lower in both serum and CSF among AD cases compared with controls [4, 13]. In sum, the literature supports VEGFA alterations over the course of the AD neuropathological cascade, but the exact association appears to depend on the tissue, isoform, and stage of disease at which the sample is acquired.

Despite the complexity and mixed evidence of VEGF associations with AD dementia, there is growing evidence that VEGF may have a neuroprotective role. For example, work from our group has indicated that higher-baseline CSF VEGFA levels are associated with slower rates of hippocampal atrophy and slower rates of cognitive decline [3], particularly among individuals with elevated levels of AD biomarkers. Mouse models of AD offer additional support for the neuroprotective role of VEGFA, as memory deficits were halted when animals were treated with VEGFA [2, 14, 15].

The challenge in better characterizing VEGF alterations during brain aging and AD dementia may be due to the large number of biological pathways differentially influenced by distinct VEGF signaling proteins. The mammalian VEGF protein family includes five ligands (VEGFA, VEGFB, VEGFC, VEGFD, and PGF [placental growth factor]), three receptors (FLT1 [also called VEGFR1], KDR [also called VEGFR2], and FLT4 [also called VEGFR3]), and two co-receptors (NRP1 and NRP2), each with multiple isoforms. These ligands and receptors interact to exert different downstream effects [1]. For example, while it has been suggested that VEGFB signaling through the FLT1 receptor can mediate neuroprotection through NMDA alterations [16, 17], KDR and FLT4 signaling have been hypothesized to mediate angiogenesis through AKT signaling pathways and activation of transcription factors [1, 18]. Comprehensive assessment of VEGF ligands and receptors in the context of cognitive aging may provide clues as to which VEGF signaling pathways are most relevant to neuroprotection.

The aim of this study was to examine associations between prefrontal cortex expression of *VEGF* ligand and receptor genes in relation to cognitive performance, AD dementia, and postmortem AD neuropathology. We hypothesized that expression of *VEGF* genes in the brain would differentially relate to cognitive performance and AD pathology.

## Materials and methods

### Participants

Data were acquired from two well-characterized cohort studies of aging and dementia. The Religious Orders Study (ROS), begun in 1994, and the Rush Memory and Aging Project (MAP), begun in 1997, involve older adults who enrolled without dementia, agreed to annual clinical evaluations and organ donation at death, and signed an Anatomical Gift Act for brain donation [19–21]. Written informed consent was obtained from participants, and research was carried out in accordance with Institutional Review Board (IRB)-approved protocols. Secondary analyses of all data were approved by the Vanderbilt University Medical Center IRB. ROSMAP data are available online at the Rush Alzheimer’s Disease Center Resource Sharing Hub (https://www.radc.rush.edu/), as well as on the Accelerating Medicines Partnership-Alzheimer’s Disease (AMP-AD) Knowledge Portal (syn3219045).

### Neuropsychological composites

Composite measures of cognition have been calculated in ROS/MAP [22]. Briefly, global cognition was derived from a total of 17 tests across five domains of cognition (episodic, semantic, and working memory, perceptual orientation, and perceptual speed). A global cognition composite was made by averaging the *z*-scores of all available tests.

### Autopsy measures of VEGF gene expression

RNA expression levels were obtained from frozen sections of the dorsolateral prefrontal cortex that were manually dissected from postmortem brain tissue. Details of RNA extraction, processing, quality control, and normalization have been published [23]. Briefly, RNA was isolated using the RNeasy lipid tissue kit (Qiagen, Valencia, CA) and was reverse transcribed and biotin-UTP labeled using the llumina^®^ TotalPrep™ RNA Amplification Kit from Ambion™ (Illumina, San Diego, CA). Expression signals were generated using the BeadStudio software suite (Illumina, San Diego, CA). Standard control and normalization methods were employed to account for technical variability, due to differences in hybridization dates. Expression levels of ten *VEGF* ligand and receptor genes with 63 available isoforms were analyzed (number of isoforms in parenthesis): *VEGFA* (14), *VEGFB* (3), *VEGFC* (2), *VEGFD* (2), *NRP1* (12), *NRP2* (13), *FLT1* (3), *FLT4* (8), *KDR* (1), and *PGF* (5). Primary analyses focused on gene count data quantified using RSEM. Reads were aligned using the Bowtie 1 package and then counted using RSEM. Secondary analyses assessed isoform abundance, which was also quantified using RSEM. Low-abundance isoforms (expressed in < 10% of the cohort) were filtered out from analyses to reduce confounding due to floor effects. Outliers (values 4 standard deviations above or below the combined sample mean) were also removed.

### Measures of neural and cerebrovascular pathology

All measures of pathology were characterized previously in ROS/MAP [19, 20]. β-amyloid and tau neuropathologies were measured in two ways: immunohistochemistry and microscopic evaluation of silver-stain cross-sections. The percentage of area occupied by β-amyloid or tau was an average of anti-Aβ and anti-phosphorylated tau immunohistochemistry evaluation from eight regions (hippocampus, angular gyrus, and entorhinal, midfrontal, inferior temporal, calcarine, anterior cingulate, and superior frontal cortices). Counts of neuritic plaques and neurofibrillary tau tangles (NFT) were evaluated on silver-stained slides from five regions (hippocampus and entorhinal, midtemporal, inferior parietal, and midfrontal cortices). These counts were entered as continuous variables and were transformed prior to analysis to better approximate a normal distribution.

TDP-43 pathology was analyzed in six regions (amygdala, hippocampus CA1, dentate gyrus, entorhinal, midtemporal, and midfrontal cortices) by staining with monoclonal antibodies to phosphorylated TDP-43 and was scored from 0 (no pathology) to 4 (presence of pathology in all regions) [24]. Measurement details for cerebral amyloid angiopathy (CAA), atherosclerosis, arteriolosclerosis, and gross- and micro-infarcts have been published [25–31]. Briefly, CAA was measured in the midfrontal, midtemporal, angular, and calcarine cortices and scored from 0 to 3, with 3 being the highest deposition level [27, 29]. Atherosclerosis was measured by visual inspection of circle of Willis vessels and given a score from 0 (no significant atherosclerosis) to 3 (over half had atherosclerosis or at least one had 75% occlusion or both) [25]. Arteriolosclerosis was measured histologically and classified into four stages, 0 indicating no changes and 3 indicating severe changes [28]. Gross infarcts were measured from nine regions (midfrontal, middle temporal, entorhinal, hippocampal, inferior parietal and anterior cingulate cortices, anterior basal ganglia, thalamus, and midbrain) by visual inspection and confirmed histologically. Micro-infarcts were examined from the same nine regions by 6-µm paraffin-embedded sections stained with hematoxylin/eosin. Both were given a binary score, indicating the presence or absence of infarcts [26, 30, 31]. In addition, total count and volume of macro-infarcts were assessed by visual inspection to quantify the extent of ischemic brain damage.

### Differential expression replication data sets

Data from two additional cohorts from the AMP-AD Knowledge Portal (syn14237651), the MayoRNAseq study (syn5550404), and the Mount Sinai Brain Bank (MSBB) study (syn3159438), were leveraged for replication of differential expression results. For the Mayo cohort, postmortem samples were collected from the temporal cortex and cerebellum, as previously described [32, 33]. For the MSBB cohort, postmortem samples were collected from the parahippocampal gyrus, frontal pole, superior temporal gyrus, and inferior frontal gyrus, as previously described [34]. Clinical diagnosis was harmonized between the studies using Braak staging, tau pathology, and cognitive scores.

### Statistical analyses

Statistical analyses were completed using R (versions 3.4.3 and 3.5.0; https://www.r-project.org/) and code is available on request from the authors. Significance was set a priori to *α* = 0.05. *P*-value corrections were performed using the false discovery rate (FDR) method. All models were restricted to individuals who had their last cognitive assessment within 2 years of death. For all analyses, each gene was run individually.

Prior to the main analyses, we assessed differences in *VEGF* expression among diagnostic groups using linear regression models, covarying for age at death and sex. In replication data sets, weighted mixed-effect linear models with sex and age of death as fixed effects and donor as a random effect were used to analyze differences in gene expression between participants with AD and controls in each study. *P*-value corrections were made using the FDR procedure, correcting for all genes analyzed (between 16,348 and 18,520 depending on the tissue). Normalization and covariate adjustments were performed for each study separately to account for differences between studies.

For the main analysis, we evaluated associations between *VEGF* expression levels in prefrontal cortex tissue with global cognition cross-sectionally (at the final visit prior to death) as well as longitudinally (over the years preceding death). Longitudinal associations were evaluated using mixed-effect regression with a gene expression × interval interaction term, with interval modeled as time in years before death. Age at death, sex, gene expression level, and interval were entered as fixed effects with the intercept and interval entered as random effects. The global cognition composite was entered as a continuous outcome. Cross-sectional associations were evaluated using linear regression covarying for age at death, sex, and interval between the last visit and death. Secondary analyses analyzed diagnostic interactions and assessed isoform abundance data to clarify the most relevant isoforms of each gene.

To remove possible effects of sample differences in cell-type composition across age and disease, proportions of neurons, microglia, oligodendrocytes, and endothelial cells were estimated using the prefrontal cortex expression of known cell-specific marker genes: *ENO2* (neurons), *CD68* (microglia), *OLIG2* (oligodendrocytes), *GFAP* (astrocytes), and *CD34* (endothelial cells). Correlations between *VEGF* genes and cell-specific markers were calculated using Pearson's R. Next, models were rerun covarying for *ENO2* in one model and again covarying for all other cell types in a second model. In addition, we residualized the association between a given gene and cell marker to create a corrected expression score and analyzed the models again.

Last, we evaluated associations between *VEGF* expression levels and AD neuropathology. Associations with continuous outcomes of β-amyloid, tau, neuritic plaques, and NFT were analyzed using linear models. Secondary analyses assessed associations between *VEGF* expression and non-AD neuropathology. Associations with multilevel categorical outcomes (CAA, atherosclerosis, arteriolosclerosis, and TDP-43) were analyzed with proportional-odds logistic regression. Associations with binary categorical outcomes were analyzed with binary logistic regression. Associations with gross-infarct count and volume were assessed using Poisson regression. All models covaried for age at death and sex.

## Results

Participant characteristics are presented in Table 1. Participants were mostly well-educated, non-Hispanic white, and had diagnoses of normal cognition, mild cognitive impairment, and AD dementia. As expected, global cognition scores differed significantly across diagnostic groups (*p* < 0.001). Participants with AD dementia had an older mean age of death compared with the other groups (*p* < 0.001).

**Table 1:** Participant characteristics

	Clinical diagnosis			Total (531)	*P*
	Normal cognition (180)	Mild cognitive impairment (148)	Alzheimer’s disease (203)		
Age of death, years	86 ± 7	89 ± 6	91 ± 6	89 ± 7	**<0.001**
Male, no. (%)	70 (39)	54 (36)	70 (34)	194 (37)	0.67
Non-Hispanic white, no. (%)	177 (98)	146 (99)	195 (96)	518 (98)	0.21
Education, years	17 ± 4	16 ± 3	17 ± 4	17 ± 4	0.59
Global cognition composite (at the last visit), z	0.14 ± 0.42	−0.49 ± 0.45	−1.85 ± 0.91	−0.80 ± 1.09	**<0.001**
Average number of visits	7.12 ± 4.04	6.93 ± 3.65	7.55 ± 3.69	7.23 ± 3.8	0.26

Boldface signifies *p* < 0.05

### Diagnostic differences in VEGF expression

**Fig. 1:**
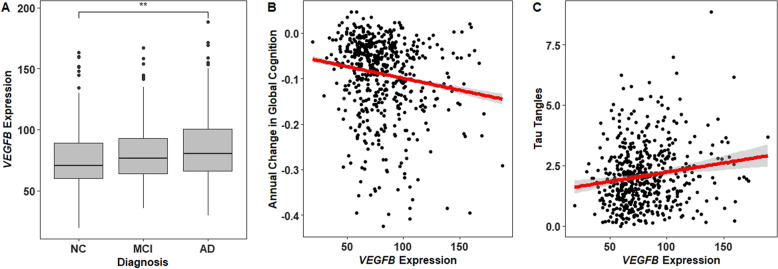
VEGFB associations with longitudinal cognition, AD dementia, and tau pathology. Prefrontal cortex expression of *VEGFB* (**a**) differed between participants with AD dementia compared with those with normal cognition, **b** was negatively associated with longitudinal global cognition, and **c** was positively associated with tau burden at autopsy. **p-value < 0.01; NC normal cognition, MCI mild cognitive impairment, AD Alzheimer’s disease

*VEGFB* was expressed at higher levels in AD dementia as compared with normal cognition participants (corrected *p* = 0.04; Fig. 1b), a finding which replicated in both the Mayo (cerebellum and temporal cortex tissues) and MSBB cohorts (parahippocampal gyrus). Differences across all three diagnostic groups for the whole *VEGF* family are presented in Supplementary Table 1. In addition, we observed nominal increased expression between participants with neuropathologically confirmed AD compared with controls in *FLT1* and *FLT4* (corrected *p* > 0.05, *p* < 0.01; Supplementary Figs. 1A and 2A), which replicated among Mayo participants in the temporal cortex for *FLT4* (corrected *p* = 0.006) and in both the cerebellum and temporal cortex for *FLT1* (corrected *p* < 0.02). See Supplementary Tables 2 and 3 for differential expression results for the whole *VEGF* family in the AMP-AD cohorts.

### Associations with cognition

Longitudinal and cross-sectional associations with global cognition are presented in Table 2. In cross-sectional analyses, increased *VEGFB* and *FLT4* expression levels were associated with worse cognition. In longitudinal analyses, higher levels of ligand genes *VEGFB* and *PGF* and receptor genes *FLT1* and *FLT4* were associated with worse cognitive trajectories (Fig. 1b, Supplementary Figs. 3A, 1B, and 2B). In sensitivity analyses covarying for diagnosis (Supplementary Table 4), all longitudinal associations remained significant (*p* ≤ 0.004), and of the two cross-sectional associations, *VEGFB* remained significant (*p* = 0.047) while *FLT4* did not (*p* = 0.391). In addition, no interactions were observed between diagnosis and gene expression in predicting cognition cross-sectionally or longitudinally after correction for multiple tests (Supplementary Table 5). In isoform sensitivity analyses (Supplementary Table 6), all four *VEGF* genes associated with cognition showed two or more isoform-specific associations comparable with the gene-level results.

**Table 2:** VEGF associations with global cognition

	Longitudinal				Cross-sectional			
Gene	*β*	SE	*P*	P.fdr	*β*	SE	*P*	P.fdr
*VEGFB*	−0.001	0.0002	5.66E−05	**0.001**	−0.006	0.002	0.001	**0.006**
*FLT4*	−0.030	0.009	4.47E−04	**0.002**	−0.207	0.078	0.008	**0.040**
*FLT1*	−0.004	0.001	0.002	**0.005**	−0.025	0.012	0.038	0.126
*PGF*	−0.010	0.003	0.002	**0.005**	−0.051	0.028	0.075	0.187
*NRP1*	0.004	0.004	0.279	0.484	0.054	0.036	0.133	0.265
*NRP2*	0.004	0.007	0.582	0.646	0.073	0.066	0.273	0.455
*VEGFC*	−0.017	0.018	0.339	0.484	−0.128	0.164	0.437	0.546
*VEGFD*	−0.006	0.015	0.693	0.693	−0.109	0.139	0.436	0.546
*VEGFA*	−0.001	0.001	0.302	0.484	−0.003	0.005	0.493	0.547
*KDR*	−0.007	0.012	0.540	0.646	0.044	0.113	0.697	0.697

Boldface signifies corrected P.fdr < 0.05

P.fdr column contains *p*-values corrected for ten tests using the false discovery rate (FDR)

Several genes were correlated with expression of cell-specific markers (see Supplementary Fig. 4 for correlation matrix). Specifically, *VEGFB, FLT4*, and *PGF* showed strong negative correlations (*r* < −0.55) with *ENO2* expression, a marker for neurons. *VEGFB* also showed a strong positive correlation (*r* = 0.59) with *OLIG2* expression, a marker for oligodendrocytes. Although cross-sectional associations were attenuated when adjusting for cell-specific markers of neurons (see Supplementary Table 7), longitudinal results remained largely unchanged, regardless of the type of cell-specific adjustment applied (see Supplementary Table 8).

### Associations with pathology

Next, we tested for associations between the four *VEGF* genes that showed significant associations with cognition (*VEGFB*, *FLT1*, *FLT4*, and *PGF*) and neuropathology. The results for AD pathology are presented in Table 3. In analyses of β-amyloid burden, we observed associations with *FLT1*, *FLT4*, and *PGF* (see Supplementary Figs. 1B, 2B, and 3B). In analyses of tau burden, we observed associations with *VEGFB*, *FLT4*, and *PGF* (Fig. 1c, Supplementary Figs. 2D and 3D). *VEGFB*, *PGF*, and *FLT4* were also associated with neuritic plaques. No associations were observed with NFT. See Supplementary Table 9 for associations between the six VEGF genes, which had no associations with cognition and AD pathology. In analyses of non-AD pathology, *VEGFB* was positively associated with atherosclerosis (*β* = 0.006, SE = 0.003, and *p* = 0.039) and arteriolosclerosis (*β* = 0.008, SE = 0.003, and *p* = 0.013; see Supplementary Table 10). VEGFB and FLT1 were associated with increased infarct count (*p* < 0.005) and volume (*p* < 0.035; see Supplementary Table 11). See Supplementary Table 12 for isoform associations with pathology.

**Table 3:** VEGF associations with AD pathology

	β-amyloid				Tau				Neuritic plaques				Neurofibrillary tangles			
Gene	*β*	SE	*P*	P.fdr	*β*	SE	*P*	P.fdr	*β*	SE	*P*	P.fdr	*β*	SE	*P*	P.fdr
*FLT1*	0.045	0.013	0.001	**0.002**	0.022	0.014	0.122	0.122	0.009	0.006	0.136	0.136	0.006	0.004	0.193	0.193
*FLT4*	0.243	0.084	0.004	**0.008**	0.228	0.091	0.013	**0.017**	0.078	0.038	0.040	0.053	0.053	0.028	0.056	0.168
*PGF*	0.060	0.031	0.049	0.066	0.107	0.033	0.001	**0.003**	0.034	0.014	0.014	**0.028**	0.015	0.010	0.126	0.168
*VEGFB*	0.002	0.002	0.190	0.190	0.007	0.002	0.001	**0.003**	0.002	0.001	0.010	**0.028**	0.001	0.001	0.113	0.168

Boldface signifies corrected P.fdr < 0.05

P.fdr column contains *p*-values corrected for four tests using the false discovery rate (FDR)

**Fig. 2:**
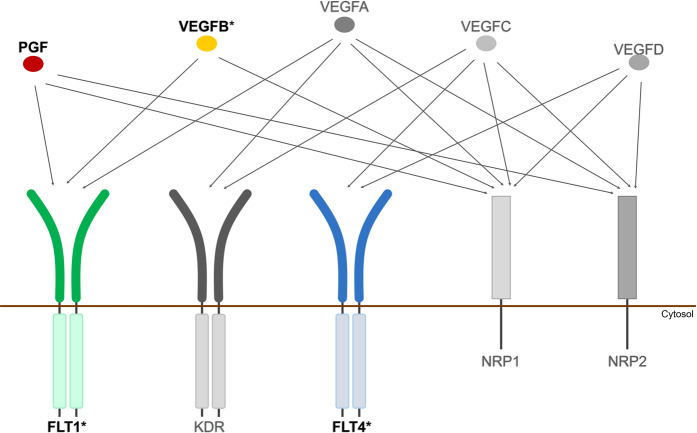
Associations in the VEGF family with cognition, AD dementia, and AD pathology. This figure presents an illustration of the VEGF family, with arrows drawn from each ligand to each receptor/co-receptor with which it binds. Gene expression levels of the ligands and receptors in color (*VEGFB*, *PGF*, *FLT4*, and *FLT1*) were associated with worsening cognitive performance prior to death and associated with amyloid or tau pathology at autopsy. Asterisk indicates genes that were expressed differently between participants with AD dementia and those with normal cognition

Taking all these results together, the model in Fig. 2 illustrates how the VEGF ligands and receptors interact, and which components are associated with cognition, AD dementia, and AD pathology.

## Discussion

Prefrontal cortex expression of multiple *VEGF* genes, including ligand genes *VEGFB* and *PGF* and receptor genes *FLT1* and *FLT4*, were associated with a more rapid rate of cognitive decline late in life, with *VEGFB* and *FLT4* also associated with cognitive scores closest to death. In addition, higher expression levels of these genes were observed among individuals with AD dementia, a finding which replicated across multiple data sets, and were associated with increased AD neuropathology at autopsy. These results suggest that alterations along VEGF signaling pathways occur with AD-related decline and are relevant to the cognitive progression of the disease.

We observed associations between prefrontal cortex expression of *FLT1* and its ligand genes *VEGFB* and *PGF* in relation to longitudinal cognitive performance, with *VEGFB* also associated with cognition at the last visit prior to death. While VEGFA-KDR signaling is the classical angiogenic pathway, FLT1 is known to have dual roles in neurogenesis and angiogenesis. Membrane-bound FLT1 signaling has been well-characterized in peripheral vascular cells where it is known to facilitate inflammatory cell recruitment [35], but the recent recognition that FLT1 may be predominantly expressed in neurons [36] and glia [37] in AD brains raises the question of unidentified roles for this receptor in neural repair processes, particularly under conditions of systemic hypoxia. For example, VEGFB has been shown to downregulate cell death-related signaling pathways in neurons through tyrosine-kinase activity of FLT1 [38, 39]. Thus, FLT1-mediated VEGFB signaling appears to promote neuroprotection (rather than acting as an angiogenic factor) in injury models of the nervous system. In support of this, we observed increased ischemic damage associated with higher expression of *VEGFB* and *FLT1*. In the present analysis, associations between *VEGFB* and faster cognitive decline may therefore reflect a downstream neural repair response to AD pathology.

FLT1 may also promote angiogenesis through PGF signaling. Although both VEGFB and PGF bind to FLT1, PGF appears to have stronger angiogenic effects compared with VEGFB, inducing neurovascular repair and the formation of perfused arterialized microvessels [40]. In our analyses, higher *PGF* expression related to worse clinical outcomes, potentially reflecting a proangiogenic state coinciding with AD pathology that may be beneficial or detrimental to brain health. However, from the present data it is difficult to determine whether the observed genetic associations are the cause or consequence of disease.

The mixed accounts of FLT1’s role in angiogenesis are partially clarified by noting the distinct roles that its isoforms play. Membrane-bound FLT1 is the signaling subtype (implicated in the VEGFB and PGF studies above) whereas soluble FLT1 is the inert decoy subtype, which potently inhibits VEFGA-induced angiogenesis by sequestering VEGFA and blocking its activity [41]. Thus, FLT1 may have additional, indirect effects on angiogenesis by regulating the bioavailability of VEGFA in the brain. Notably, when evaluating *FLT1* isoforms in the present analysis, only isoforms encoding membrane-bound FLT1 proteins showed associations with cognition and β-amyloid and tau burden, suggesting that the membrane-bound form of *FLT1* may be particularly relevant to AD dementia. The isoform-specific effects of FLT1 at the gene and protein level highlight the need for a more comprehensive assessment of VEGF ligand and receptor activity during aging and disease. Our observations support FLT1 as a signaling hub for AD-related alterations.

Given the cross-sectional nature of the gene expression measurement in the present analyses, it is unclear whether the observed effects are a cause or consequence of neuropathology. Both γ-secretase [42] and β-secretase [43] (which cleave the amyloid precursor protein) are known regulators of FLT1, particularly membrane-bound forms of FLT1 [44], suggesting that there is a common upstream regulator of both AD pathology and *VEGF* receptor genes. However, FLT1 contains structural hypoxic response elements which enable it to be directly upregulated in hypoxia-ischemia [45], leaving open the possibility that the observed gene expression differences are in response to injury downstream of pathology. Regardless of the causal or non-causal role, the present results suggest there are changes in the VEGFB*-*FLT1 signaling axis during the development AD pathology which are relevant to the cognitive progression of the disease.

In addition to *FLT1*, we observed comparable associations with *FLT4*, whereby higher levels of prefrontal cortex expression were (1) observed among participants with AD dementia compared with those with normal cognition, (2) associated with faster rates of cognitive decline in the years preceding death, and (3) associated with greater amyloid and tau burden at autopsy. Similar to FLT1, FLT4 contains structural elements that are directly responsive to hypoxia [46], suggesting a potential common pathway. FLT4 appears to be required for adult neurogenesis [47] and the upregulation of lymphogenesis, but not angiogenesis in the brain [48]. Given these beneficial effects, the negative association of *FLT4* observed here may reflect a cellular response to AD neuropathology rather than acting as a driver of neuropathology and neural injury.

This study has multiple strengths, including the well-characterized cohorts assessed, the measurement of *VEGF* ligand and receptor genes in postmortem brain tissue, and the availability of comprehensive longitudinal cognitive data. However, the measurement of gene expression in postmortem brain tissue precludes interpretation about causality or directionality from our analyses and leaves open the possibility that observed effects could be a cause, a consequence, or simply a co-occurrence with disease. Further, brain homogenates were leveraged for RNA analysis and include cell-type differences across individuals that could confound results. We attempted to control for these effects by adjusting for cell-type-specific gene expression markers and the results from the adjusted models did not change the interpretation of our main findings. However, such statistical adjustments may be insufficient to fully correct for cell-specific effects. In addition, only binary variables of the presence or absence of micro-infarcts were available for analysis, and we had no biochemical measure of hypoperfusion in the brain, limiting our ability to draw definitive conclusions about all types of ischemic damage in the brain. Finally, the sample evaluated included a highly educated, homogenous cohort of primarily European ancestry that may not generalize to other populations.

In summary, we examined associations across the *VEGF* family of genes with cognition, AD dementia, and AD pathology, and identified genes in the FLT4 and FLT1 signaling pathways as being particularly relevant. Future work will explore how VEGF family protein expression associates with cognition and AD pathology to better understand how changes in *VEGF* transcription and translation relate to neurodegenerative disease.

## Supplementary information

Supplementary Figures

Supplementary Tables 1-12
