# Supplementary material for: Brain expression of the vascular endothelial growth factor gene family in cognitive aging and alzheimer’s disease
Source: Mol Psychiatry. 2019 Jul 22;26(3):888–96. doi: 10.1038/s41380-019-0458-5 (PMC6980445; doi:10.1038/s41380-019-0458-5)
Supplement: Supplementary file 1 — Supplementary Figures [file 41380_2019_458_MOESM1_ESM.docx]

# Supplementary Figure 1. Associations with FLT1 expression


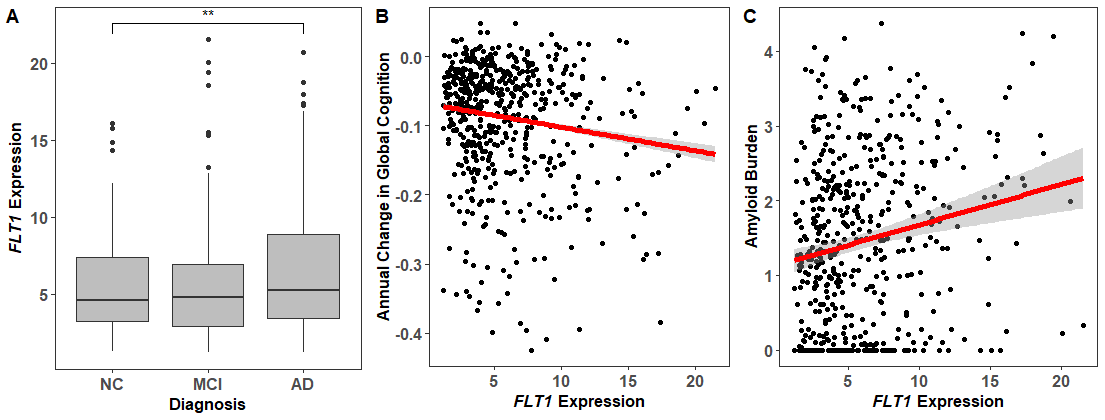


FLT1 expression associates with (A) clinical diagnosis, (B) longitudinal cognition, and (C) amyloid pathology. Note: ** indicates p-value < 0.05; NC = normal cognition, MCI = mild cognitive impairment, AD = Alzheimer’s disease.

# Supplementary Figure 2. Associations with *FLT4* expression


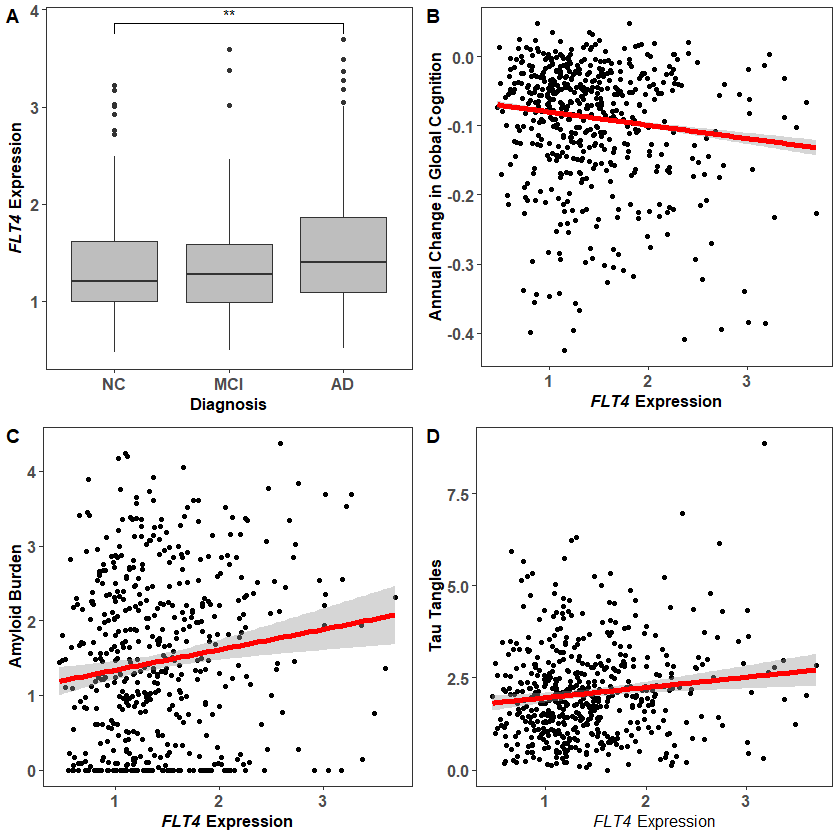


FLT4 expression associates with (A) clinical diagnosis, (B) longitudinal cognition, and (C) amyloid and (D) tau pathology. Note: ** indicates p-value < 0.05; NC = normal cognition, MCI = mild cognitive impairment, AD = Alzheimer’s disease.

# Supplementary Figure 3. Associations with *PGF* expression


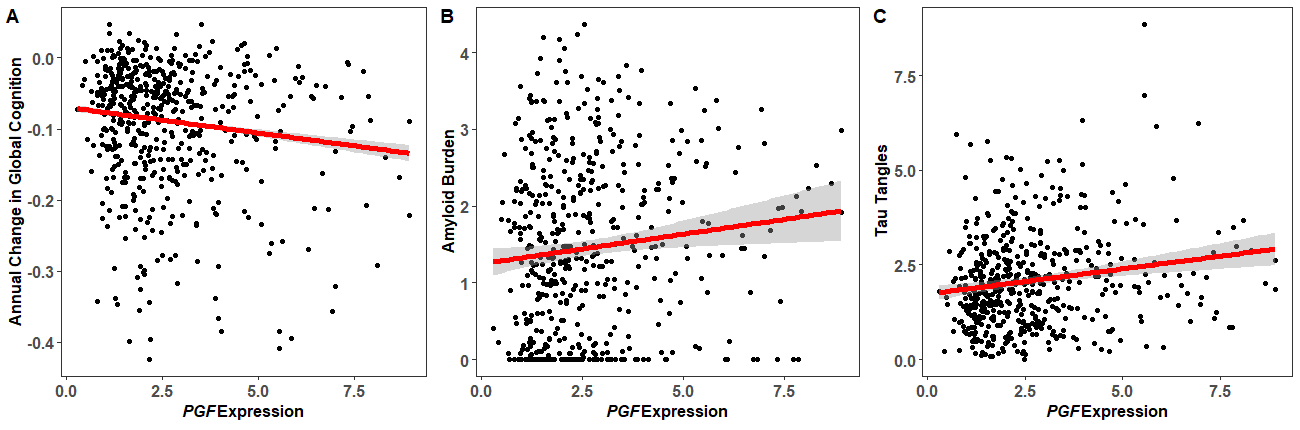


PGF expression associates with (A) longitudinal cognition as well as (B) amyloid and (C) tau pathology.

# Supplementary Figure 4. Correlations between *VEGF* expression and expression of cell-type markers


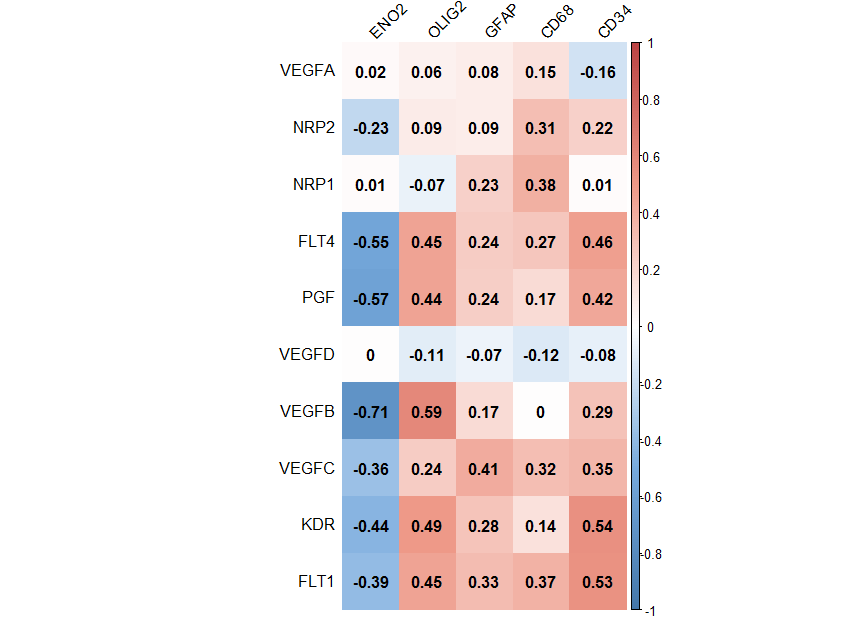


Correlations between expression of VEGF genes and cell-type markers (ENO2 [neurons], CD68 [microglia], OLIG2 [oligodendrocytes], GFAP [astrocytes], and CD34 [endothelial cells]). Correlations were calculated using Pearson’s R and range from -1 (blue color) to 1 (red color).
