# Supplementary material for: Brain expression of the vascular endothelial growth factor gene family in cognitive aging and alzheimer’s disease
Source: Mol Psychiatry. 2019 Jul 22;26(3):888–96. doi: 10.1038/s41380-019-0458-5 (PMC6980445; doi:10.1038/s41380-019-0458-5)
Supplement: Supplementary file 2 — Supplementary Tables 1-12 [file 41380_2019_458_MOESM2_ESM.docx]

# Supplementary Table 1. VEGF Expression by Diagnostic Category

Note: Differences across diagnostic groups were assessed using linear regression covarying for age at death and sex and summarized using an ANOVA. Differences between participants with AD and normal cognition are the AD term in the linear regression model.

| **gene** | **Normal Cognition (N=180)** | **Mild Cognitive Impairment (N=148)** | **Alzheimer’s Disease (N=203)** | **P - across diagnoses** | **p.fdr - across diagnoses** | **P - between NC and AD** | **p.fdr - between NC and AD** |
| --- | --- | --- | --- | --- | --- | --- | --- |
| *VEGFB* | 76.51 ± 25.92 | 80.8 ± 24.69 | 85.03 ± 26.65 | 0.016 | 0.079 | 0.0040 | **0.0400** |
| *FLT4* | 1.37 ± 0.57 | 1.33 ± 0.51 | 1.53 ± 0.63 | 0.006 | 0.055 | 0.0175 | 0.0873 |
| *FLT1* | 5.45 ± 3.07 | 5.64 ± 3.83 | 6.61 ± 4.19 | 0.050 | 0.168 | 0.0317 | 0.1056 |
| *PGF* | 2.45 ± 1.54 | 2.55 ± 1.49 | 2.84 ± 1.72 | 0.191 | 0.424 | 0.0938 | 0.2069 |
| *NRP1* | 4.14 ± 1.15 | 4.11 ± 1.35 | 3.93 ± 1.24 | 0.212 | 0.424 | 0.1034 | 0.2069 |
| *VEGFC* | 0.54 ± 0.25 | 0.6 ± 0.29 | 0.6 ± 0.28 | 0.271 | 0.452 | 0.1961 | 0.3269 |
| *VEGFA* | 12.9 ± 9.55 | 13.68 ± 9.47 | 13.81 ± 10.09 | 0.766 | 0.851 | 0.5087 | 0.7267 |
| *KDR* | 0.93 ± 0.38 | 0.92 ± 0.38 | 0.97 ± 0.43 | 0.583 | 0.794 | 0.7323 | 0.7378 |
| *VEGFD* | 0.99 ± 0.33 | 0.99 ± 0.3 | 0.98 ± 0.33 | 0.941 | 0.941 | 0.7378 | 0.7378 |
| *NRP2* | 2.63 ± 0.66 | 2.58 ± 0.69 | 2.64 ± 0.68 | 0.635 | 0.794 | 0.6298 | 0.7378 |

# Supplementary Table 2. Differential *VEGF* Expression (Mayo)

Note: Abbreviations: TCX = temporal cortex, CBE = cerebellum; Adjusted p column contains p values corrected using the FDR procedure.

| **Gene** | **Number Cases/Controls** | **T Statistic** | **P** | **Adjusted P** |
| --- | --- | --- | --- | --- |
| Cerebellum (CBE) | | | | |
| *VEGFD* | 79/72 | -3.867 | 1.25E-04 | **0.001** |
| *FLT1* | 79/72 | 3.036 | 0.0025 | **0.011** |
| *VEGFB* | 79/72 | -2.561 | 0.011 | **0.034** |
| *NRP2* | 79/72 | 2.347 | 0.019 | 0.054 |
| *PGF* | 79/72 | 2.120 | 0.035 | 0.083 |
| *FLT4* | 79/72 | 1.949 | 0.052 | 0.116 |
| *NRP1* | 79/72 | 1.823 | 0.069 | 0.145 |
| *VEGFA* | 79/72 | 0.964 | 0.336 | 0.470 |
| *KDR* | 79/72 | 0.783 | 0.434 | 0.564 |
| *VEGFC* | 79/72 | -0.338 | 0.735 | 0.817 |
| Temporal cortex (TCX) | | | | |
| *FLT1* | 80/71 | 5.289 | 1.87E-07 | **4.59E-06** |
| *VEGFC* | 80/71 | 4.562 | 6.46E-06 | **7.47E-05** |
| *VEGFB* | 80/71 | 4.033 | 6.40E-05 | **4.77E-04** |
| *FLT4* | 80/71 | 3.194 | 0.0015 | **0.006** |
| *PGF* | 80/71 | 3.071 | 0.0023 | **0.008** |
| *NRP1* | 80/71 | 2.076 | 0.038 | 0.085 |
| *VEGFA* | 80/71 | 1.694 | 0.091 | 0.164 |
| *KDR* | 80/71 | 1.474 | 0.141 | 0.232 |
| *VEGFD* | 80/71 | 1.361 | 0.174 | 0.273 |
| *NRP2* | 80/71 | 1.175 | 0.240 | 0.351 |

# Supplementary Table 3. Differential *VEGF* Expression (Mount Sinai)

Note: Genes in red were differentially expressed in the main analysis dataset.

| **Gene** | **Number Cases/Controls** | **T Statistic** | **P Value** | **Adjusted P** |
| --- | --- | --- | --- | --- |
| Frontal pole (FP) | | | | |
| *NRP1* | 90/45 | -2.985445 | 0.00293 | 0.099 |
| *FLT1* | 90/45 | -2.002868 | 0.0456 | 0.333 |
| *KDR* | 90/45 | -0.6838625 | 0.494 | 0.635 |
| *VEGFA* | 90/45 | -1.091289 | 0.276 | 0.661 |
| *NRP2* | 90/45 | 0.8123284 | 0.417 | 0.701 |
| *FLT4* | 90/45 | -0.5603271 | 0.575 | 0.855 |
| *PGF* | 90/45 | 0.35268 | 0.724 | 0.919 |
| *VEGFB* | 90/45 | 0.1769846 | 0.860 | 0.963 |
| Inferior frontal gyrus (IFG) | | | | |
| *PGF* | 79/37 | 2.304538 | 0.0215 | 0.071 |
| *NRP1* | 79/37 | -2.496798 | 0.0128 | 0.190 |
| *FLT4* | 79/37 | 2.482206 | 0.0133 | 0.193 |
| *VEGFB* | 79/37 | 2.468068 | 0.0138 | 0.195 |
| *KDR* | 79/37 | -0.879446 | 0.379 | 0.686 |
| *VEGFA* | 79/37 | -0.87825 | 0.380 | 0.687 |
| *NRP2* | 79/37 | 0.2713592 | 0.786 | 0.913 |
| *FLT1* | 79/37 | -0.2117326 | 0.832 | 0.934 |
| Parahippocampal gyrus (PHG) | | | | |
| *VEGFB* | 65/38 | 4.539522 | 6.63E-06 | **3.68E-04** |
| *KDR* | 65/38 | 2.607833 | 0.00930 | **0.039** |
| *PGF* | 65/38 | 2.529947 | 0.0116 | **0.045** |
| *NRP2* | 65/38 | 2.384424 | 0.0174 | 0.059 |
| *FLT4* | 65/38 | 2.291148 | 0.0223 | 0.230 |
| *NRP1* | 65/38 | 0.7379016 | 0.461 | 0.603 |
| *FLT1* | 65/38 | 0.9071133 | 0.365 | 0.636 |
| *VEGFA* | 65/38 | -0.6816028 | 0.496 | 0.810 |
| Superior temporal gyrus (STG) | | | | |
| *NRP2* | 85/37 | 2.725703 | 0.00658 | 0.109 |
| *FLT4* | 85/37 | 2.179222 | 0.0297 | 0.201 |
| *VEGFA* | 85/37 | -1.702991 | 0.0890 | 0.330 |
| *PGF* | 85/37 | 1.208014 | 0.227 | 0.513 |
| *NRP1* | 85/37 | -0.9167883 | 0.360 | 0.513 |
| *FLT1* | 85/37 | 0.8857153 | 0.376 | 0.648 |
| *VEGFB* | 85/37 | 0.7682337 | 0.443 | 0.686 |
| *KDR* | 85/37 | -0.7992712 | 0.424 | 0.766 |

# Supplementary Table 4. VEGF associations with global cognition, covarying for diagnosis

Note: Longitudinal models used mixed-effects regression with age at death, sex, gene expression level, and interval entered as fixed effects and the intercept and interval entered as random effects. Cross-sectional models were evaluated using linear regression covarying for age at death, sex, and interval between last visit and death.

|  | **Cross-Sectional Results** | | | | **Cross-Sectional Results Covarying for Diagnosis** | | | | **Longitudinal Results** | | | | **Longitudinal Results Covarying for Diagnosis** | | | |
| --- | --- | --- | --- | --- | --- | --- | --- | --- | --- | --- | --- | --- | --- | --- | --- | --- |
| **gene** | **β** | **SE** | **DF** | **P** | **β** | **SE** | **DF** | **P** | **β** | **SE** | **DF** | **P** | **β** | **SE** | **DF** | **P** |
| *VEGFB* | -0.006 | 0.002 | 524 | **0.001** | -0.002 | 0.001 | 522 | **0.047** | -0.001 | 0.0002 | 3297 | **5.66E-05** | -0.001 | 0.0002 | 3297 | **1.79E-04** |
| *FLT4* | -0.207 | 0.078 | 522 | **0.008** | -0.043 | 0.050 | 520 | 0.391 | -0.030 | 0.009 | 3287 | **4.47E-04** | -0.027 | 0.009 | 3287 | **0.002** |
| *FLT1* | -0.025 | 0.012 | 523 | 0.038 | -0.004 | 0.008 | 521 | 0.625 | -0.004 | 0.001 | 3288 | **0.002** | -0.004 | 0.001 | 3288 | **0.003** |
| *PGF* | -0.051 | 0.028 | 522 | 0.075 | -0.014 | 0.018 | 520 | 0.435 | -0.010 | 0.003 | 3295 | **0.002** | -0.009 | 0.003 | 3295 | **0.004** |
| *NRP1* | 0.054 | 0.036 | 523 | 0.133 | 0.004 | 0.023 | 521 | 0.846 | 0.004 | 0.004 | 3273 | 0.279 | 0.004 | 0.004 | 3273 | 0.283 |
| *NRP2* | 0.073 | 0.066 | 524 | 0.273 | 0.061 | 0.042 | 522 | 0.152 | 0.004 | 0.007 | 3284 | 0.582 | 0.005 | 0.008 | 3284 | 0.495 |
| *VEGFD* | -0.109 | 0.139 | 525 | 0.436 | -0.072 | 0.089 | 523 | 0.420 | -0.006 | 0.015 | 3303 | 0.693 | -0.003 | 0.016 | 3303 | 0.838 |
| *VEGFC* | -0.128 | 0.164 | 523 | 0.437 | -0.008 | 0.105 | 521 | 0.938 | -0.017 | 0.018 | 3284 | 0.339 | -0.013 | 0.019 | 3284 | 0.479 |
| *VEGFA* | -0.003 | 0.005 | 523 | 0.493 | -0.001 | 0.003 | 521 | 0.699 | -0.001 | 0.001 | 3293 | 0.302 | -0.001 | 0.001 | 3293 | 0.319 |
| *KDR* | 0.044 | 0.113 | 525 | 0.697 | 0.089 | 0.072 | 523 | 0.216 | -0.007 | 0.012 | 3306 | 0.540 | -0.007 | 0.013 | 3306 | 0.591 |

# Supplementary Table 5. VEGF and diagnosis interaction in predicting global cognition

|  | **Cross-sectional** | | | | **Longitudinal** | | | |
| --- | --- | --- | --- | --- | --- | --- | --- | --- |
| **gene** | **DF** | **Chisq** | **p** | **p.fdr** | **DF** | **Chisq** | **p** | **p.fdr** |
| *VEGFB* | 2 | 0.371 | 0.647 | 0.853 | 2 | 6.566 | 0.038 | 0.264 |
| *FLT4* | 2 | 0.773 | 0.405 | 0.810 | 2 | 5.885 | 0.053 | 0.264 |
| *PGF* | 2 | 0.924 | 0.340 | 0.810 | 2 | 4.623 | 0.099 | 0.330 |
| *NRP1* | 2 | 0.075 | 0.916 | 0.916 | 2 | 3.978 | 0.137 | 0.342 |
| *KDR* | 2 | 0.850 | 0.372 | 0.810 | 2 | 2.674 | 0.263 | 0.525 |
| *VEGFD* | 2 | 0.358 | 0.660 | 0.853 | 2 | 1.589 | 0.452 | 0.753 |
| *VEGFC* | 2 | 0.278 | 0.724 | 0.853 | 2 | 0.921 | 0.631 | 0.811 |
| *FLT1* | 2 | 2.254 | 0.071 | 0.649 | 2 | 0.865 | 0.649 | 0.811 |
| *VEGFA* | 2 | 1.756 | 0.130 | 0.649 | 2 | 0.581 | 0.748 | 0.831 |
| *NRP2* | 2 | 0.227 | 0.767 | 0.853 | 2 | 0.286 | 0.867 | 0.867 |

# Supplementary Table 6. VEGF isoform associations with cognition

Note: Longitudinal models used mixed-effects regression with age at death, sex, isoform expression, and interval entered as fixed effects and intercept and interval entered as random effects. Cross-sectional models were evaluated using linear regression covarying for age at death, sex, and interval between last visit and death.

|  |  | **Cross-sectional** | | | **Longitudinal** | | |
| --- | --- | --- | --- | --- | --- | --- | --- |
| **isoform** | **protein-coding** | **β** | **SE** | **P** | **β** | **SE** | **P** |
| *FLT4-207* | no | -1.065 | 0.343 | **0.002** | -0.163 | 0.038 | **1.73E-05** |
| *VEGFB-202* | yes | -0.028 | 0.009 | **0.001** | -0.004 | 0.001 | **3.03E-05** |
| *FLT4-201* | yes | -0.382 | 0.127 | **0.003** | -0.053 | 0.014 | **1.12E-04** |
| *VEGFB-201* | yes | -0.009 | 0.004 | **0.015** | -0.001 | 0.0004 | **0.001** |
| *FLT1-201* | yes | -0.035 | 0.015 | **0.02** | -0.005 | 0.002 | **0.003** |
| *PGF-203* | yes | -0.074 | 0.038 | 0.05 | -0.011 | 0.004 | **0.005** |
| *FLT1-204* | yes | -0.52 | 0.318 | 0.103 | -0.09 | 0.034 | **0.008** |
| *PGF-205* | no | -0.704 | 0.471 | 0.136 | -0.132 | 0.052 | **0.011** |
| *PGF-206* | yes | -0.095 | 0.08 | 0.234 | -0.02 | 0.008 | **0.018** |
| *FLT4-202* | yes | -1.149 | 0.772 | 0.137 | -0.196 | 0.084 | **0.019** |
| *VEGFB-204* | no | -0.004 | 0.002 | **0.043** | -0.001 | 0.0002 | **0.022** |
| *PGF-201* | yes | -0.44 | 0.199 | **0.027** | -0.032 | 0.022 | 0.138 |
| *FLT4-203* | no | 5.321 | 2.907 | 0.068 | 0.401 | 0.34 | 0.238 |
| *FLT4-211* | no | 0.257 | 0.407 | 0.528 | 0.043 | 0.044 | 0.33 |
| *FLT4-205* | no | -0.283 | 0.288 | 0.326 | -0.03 | 0.032 | 0.344 |
| *FLT4-210* | no | -1.281 | 1.063 | 0.229 | -0.113 | 0.12 | 0.347 |
| *PGF-207* | no | -0.695 | 0.669 | 0.3 | -0.062 | 0.071 | 0.385 |
| *FLT4-208* | no | -0.077 | 0.322 | 0.812 | -0.014 | 0.035 | 0.696 |
| *FLT1-202* | yes | 0.008 | 0.025 | 0.744 | -0.001 | 0.003 | 0.764 |

# Supplementary Table 7. Cross-sectional VEGF associations with global cognition adjusted for cell-type effects

Note: ENO2 was used as the marker for neurons, CD68 for microglia, OLIG2 for oligodendrocytes, GFAP for astrocytes, and CD34 for endothelial cells.

## Part 1: Correction for ENO2 levels

|  | **Cross-sectional Results** | | | **Cross-sectional Results - ENO2 Covariate** | | | **Cross-sectional Results - Adjusted for ENO2** | | |
| --- | --- | --- | --- | --- | --- | --- | --- | --- | --- |
| **gene** | **β** | **SE** | **P** | **β** | **SE** | **P** | **β** | **SE** | **P** |
| *VEGFB* | -0.006 | 0.002 | **0.001** | -0.0040 | 0.0025 | 0.1101 | -0.0046 | 0.0025 | 0.0702 |
| *FLT4* | -0.207 | 0.078 | **0.008** | -0.1168 | 0.0917 | 0.2032 | -0.1268 | 0.0923 | 0.1701 |
| *FLT1* | -0.025 | 0.012 | **0.038** | -0.0116 | 0.0133 | 0.3830 | -0.0171 | 0.0132 | 0.1952 |
| *PGF* | -0.051 | 0.028 | 0.075 | -0.0068 | 0.0339 | 0.8424 | -0.0097 | 0.0342 | 0.7759 |
| *NRP1* | 0.054 | 0.036 | 0.133 | 0.0496 | 0.0358 | 0.1663 | 0.0470 | 0.0361 | 0.1929 |
| *NRP2* | 0.073 | 0.066 | 0.273 | 0.1287 | 0.0676 | 0.0574 | 0.1220 | 0.0681 | 0.0740 |
| *VEGFD* | -0.109 | 0.139 | 0.436 | -0.1248 | 0.1381 | 0.3665 | -0.0830 | 0.1386 | 0.5497 |
| *VEGFC* | -0.128 | 0.164 | 0.437 | 0.0586 | 0.1743 | 0.7368 | 0.0643 | 0.1758 | 0.7148 |
| *VEGFA* | -0.003 | 0.005 | 0.493 | -0.0024 | 0.0046 | 0.5945 | -0.0035 | 0.0046 | 0.4472 |
| *KDR* | 0.044 | 0.113 | 0.697 | 0.2252 | 0.1226 | 0.0668 | 0.1896 | 0.1232 | 0.1243 |

## Part 2: Adjusting for expression of other cell-type markers

|  | **Cross-sectional Results** | | | **Cross-sectional Results - OLIG2, GFAP, CD68, and CD34 Covariates** | | | **Cross-sectional Results - Adjusted for OLIG2** | | | **Cross-sectional Results - Adjusted for GFAP** | | | **Cross-sectional Results - Adjusted for CD68** | | | **Cross-sectional Results - Adjusted for CD34** | | |
| --- | --- | --- | --- | --- | --- | --- | --- | --- | --- | --- | --- | --- | --- | --- | --- | --- | --- | --- |
| **gene** | **β** | **SE** | **P** | **β** | **SE** | **P** | **Beta** | **SE** | **P** | **β** | **SE** | **P** | **β** | **SE** | **P** | **β** | **SE** | **P** |
| *VEGFB* | -0.006 | 0.002 | **0.001** | -0.006 | 0.002 | **0.006** | -0.006 | 0.002 | **0.003** | -0.006 | 0.002 | **0.001** | -0.006 | 0.002 | **0.001** | -0.006 | 0.002 | **0.002** |
| *FLT4* | -0.207 | 0.078 | **0.008** | -0.163 | 0.098 | 0.098 | -0.183 | 0.086 | **0.034** | -0.159 | 0.079 | **0.044** | -0.236 | 0.082 | **0.004** | -0.189 | 0.089 | **0.033** |
| *FLT1* | -0.025 | 0.012 | **0.038** | -0.015 | 0.016 | 0.345 | -0.023 | 0.014 | 0.089 | -0.017 | 0.012 | 0.158 | -0.029 | 0.013 | **0.023** | -0.020 | 0.014 | 0.153 |
| *PGF* | -0.051 | 0.028 | 0.075 | -0.021 | 0.034 | 0.544 | -0.040 | 0.031 | 0.209 | -0.030 | 0.029 | 0.299 | -0.056 | 0.029 | 0.058 | -0.038 | 0.031 | 0.221 |
| *NRP1* | 0.054 | 0.036 | 0.133 | 0.055 | 0.039 | 0.157 | 0.047 | 0.036 | 0.200 | 0.070 | 0.038 | 0.064 | 0.060 | 0.038 | 0.113 | 0.051 | 0.036 | 0.160 |
| *NRP2* | 0.073 | 0.066 | 0.273 | 0.101 | 0.074 | 0.174 | 0.092 | 0.067 | 0.173 | 0.084 | 0.067 | 0.211 | 0.073 | 0.072 | 0.311 | 0.107 | 0.069 | 0.122 |
| *VEGFD* | -0.109 | 0.139 | 0.436 | -0.167 | 0.143 | 0.243 | -0.095 | 0.139 | 0.494 | -0.123 | 0.140 | 0.377 | -0.084 | 0.141 | 0.549 | -0.116 | 0.139 | 0.406 |
| *VEGFC* | -0.128 | 0.164 | 0.437 | 0.085 | 0.192 | 0.658 | -0.057 | 0.171 | 0.739 | 0.006 | 0.174 | 0.973 | -0.159 | 0.172 | 0.356 | 0.051 | 0.177 | 0.775 |
| *VEGFA* | -0.003 | 0.005 | 0.493 | -0.005 | 0.005 | 0.276 | -0.004 | 0.005 | 0.400 | -0.002 | 0.005 | 0.719 | -0.004 | 0.005 | 0.353 | -0.005 | 0.005 | 0.267 |
| *KDR* | 0.044 | 0.113 | 0.697 | 0.348 | 0.140 | **0.013** | 0.129 | 0.125 | 0.304 | 0.101 | 0.115 | 0.378 | 0.050 | 0.114 | 0.658 | 0.262 | 0.132 | **0.048** |

# Supplementary Table 8. Longitudinal VEGF associations with global cognition adjusted for cell-type effects

Note: ENO2 was used as the marker for neurons, CD68 for microglia, OLIG2 for oligodendrocytes, GFAP for astrocytes, and CD34 for endothelial cells.

## Part 1: Adjusting for ENO2 expression

|  | **Longitudinal Results** | | | **Longitudinal Results - *ENO2 Covariate*** | | | **Longitudinal Results - Adjusted for *ENO2*** | | |
| --- | --- | --- | --- | --- | --- | --- | --- | --- | --- |
| **gene** | **β** | **SE** | **P** | **β** | **SE** | **P** | **β** | **SE** | **P** |
| *VEGFB* | -0.001 | 0.000 | **5.66E-05** | -0.001 | 0.000 | **6.39E-05** | -0.001 | 0.000 | **0.020** |
| *FLT4* | -0.030 | 0.009 | **4.47E-04** | -0.030 | 0.009 | **4.46E-04** | -0.023 | 0.010 | **0.026** |
| *FLT1* | -0.004 | 0.001 | **0.002** | -0.004 | 0.001 | **0.002** | -0.003 | 0.001 | **0.018** |
| *PGF* | -0.010 | 0.003 | **0.002** | -0.010 | 0.003 | **0.002** | -0.006 | 0.004 | 0.095 |
| *NRP1* | 0.004 | 0.004 | 0.279 | 0.004 | 0.004 | 0.279 | 0.003 | 0.004 | 0.393 |
| *VEGFA* | -0.001 | 0.001 | 0.302 | -0.001 | 0.001 | 0.304 | -0.001 | 0.001 | 0.265 |
| *VEGFC* | -0.017 | 0.018 | 0.339 | -0.017 | 0.018 | 0.344 | 0.006 | 0.019 | 0.762 |
| *KDR* | -0.007 | 0.012 | 0.540 | -0.008 | 0.012 | 0.533 | 0.007 | 0.013 | 0.591 |
| *NRP2* | 0.004 | 0.007 | 0.582 | 0.004 | 0.007 | 0.589 | 0.010 | 0.008 | 0.191 |
| *VEGFD* | -0.006 | 0.015 | 0.693 | -0.006 | 0.015 | 0.688 | -0.003 | 0.015 | 0.847 |

## Part 2: Adjusting for expression of other cell-type markers

|  | **Longitudinal Results** | | | **Longitudinal Results - *OLIG2, GFAP, CD68, and CD34 Covariates*** | | | **Longitudinal Results - Adjusted for *OLIG2*** | | | **Longitudinal Results - Adjusted for *GFAP*** | | | **Longitudinal Results - Adjusted for *CD68*** | | | **Longitudinal Results - Adjusted for *CD34*** | | |
| --- | --- | --- | --- | --- | --- | --- | --- | --- | --- | --- | --- | --- | --- | --- | --- | --- | --- | --- |
| **gene** | **β** | **SE** | **P** | **β** | **SE** | **P** | **Beta** | **SE** | **P** | **β** | **SE** | **P** | **β** | **SE** | **P** | **β** | **SE** | **P** |
| *VEGFB* | -0.001 | 0.000 | **5.66E-05** | -0.001 | 0.000 | **1.33E-04** | -0.001 | 0.000 | **0.002** | -0.001 | 0.000 | **1.15E-04** | -0.001 | 0.000 | **0.000** | -0.001 | 0.000 | **0.001** |
| *FLT4* | -0.030 | 0.009 | **4.47E-04** | -0.029 | 0.009 | **0.001** | -0.026 | 0.009 | **0.006** | -0.023 | 0.009 | **0.008** | -0.030 | 0.009 | **0.001** | -0.025 | 0.010 | **0.010** |
| *FLT1* | -0.004 | 0.001 | **0.002** | -0.004 | 0.001 | **0.004** | -0.004 | 0.001 | **0.007** | -0.003 | 0.001 | **0.023** | -0.004 | 0.001 | **0.002** | -0.003 | 0.002 | 0.061 |
| *PGF* | -0.010 | 0.003 | **0.002** | -0.010 | 0.003 | **0.002** | -0.008 | 0.003 | **0.016** | -0.007 | 0.003 | **0.022** | -0.010 | 0.003 | **0.002** | -0.007 | 0.003 | **0.040** |
| *NRP1* | 0.004 | 0.004 | 0.279 | 0.003 | 0.004 | 0.459 | 0.003 | 0.004 | 0.454 | 0.008 | 0.004 | 0.069 | 0.006 | 0.004 | 0.138 | 0.003 | 0.004 | 0.385 |
| *VEGFA* | -0.001 | 0.001 | 0.302 | -0.001 | 0.001 | 0.208 | -0.001 | 0.001 | 0.212 | 0.000 | 0.001 | 0.608 | 0.000 | 0.001 | 0.347 | -0.001 | 0.001 | 0.061 |
| *VEGFC* | -0.017 | 0.018 | 0.339 | -0.014 | 0.018 | 0.450 | -0.006 | 0.019 | 0.732 | 0.007 | 0.019 | 0.705 | -0.015 | 0.019 | 0.420 | 0.017 | 0.019 | 0.390 |
| *KDR* | -0.007 | 0.012 | 0.540 | -0.004 | 0.012 | 0.750 | 0.002 | 0.013 | 0.894 | 0.001 | 0.012 | 0.910 | -0.006 | 0.012 | 0.594 | 0.028 | 0.014 | **0.049** |
| *NRP2* | 0.004 | 0.007 | 0.582 | 0.004 | 0.008 | 0.598 | 0.007 | 0.007 | 0.341 | 0.007 | 0.007 | 0.370 | 0.007 | 0.008 | 0.367 | 0.010 | 0.008 | 0.170 |
| *VEGFD* | -0.006 | 0.015 | 0.693 | -0.009 | 0.016 | 0.558 | -0.004 | 0.015 | 0.803 | -0.006 | 0.015 | 0.700 | -0.006 | 0.015 | 0.688 | -0.007 | 0.015 | 0.642 |

# Supplementary Table 9. VEGF associations with AD pathology (genes without associations to cognition)

Note: Used sqare root of continuous variables (amyloid, tangles, nft, neuritic plaques)

|  | **Amyloid** | | | **Tangles** | | | **NP** | | | **NFT** | | |
| --- | --- | --- | --- | --- | --- | --- | --- | --- | --- | --- | --- | --- |
| **gene** | **β** | **SE** | **P** | **β** | **SE** | **P** | **β** | **SE** | **P** | **β** | **SE** | **P** |
| VEGFD | 0.508 | 0.150 | **0.001** | 0.249 | 0.164 | 0.129 | 0.157 | 0.068 | **0.021** | 0.0953 | 0.0494 | 0.0542 |
| KDR | 0.308 | 0.122 | **0.012** | -0.129 | 0.133 | 0.333 | 0.021 | 0.055 | 0.706 | -0.0203 | 0.0402 | 0.6141 |
| VEGFC | 0.372 | 0.178 | **0.037** | -0.014 | 0.194 | 0.942 | -0.050 | 0.080 | 0.539 | 0.0288 | 0.0586 | 0.6235 |
| VEGFA | 0.005 | 0.005 | 0.321 | 0.001 | 0.005 | 0.835 | 0.000 | 0.002 | 0.863 | 0.0007 | 0.0016 | 0.6692 |
| NRP1 | 0.027 | 0.039 | 0.498 | -0.119 | 0.043 | **0.005** | -0.043 | 0.018 | **0.015** | -0.0310 | 0.0128 | **0.0159** |
| NRP2 | 0.034 | 0.072 | 0.636 | -0.065 | 0.078 | 0.408 | -0.030 | 0.033 | 0.356 | -0.0384 | 0.0236 | 0.1047 |

# Supplementary Table 10. VEGF associations with pathology

Note: Rows in red text indicate genes with significant associations with cognition.

*Part 1*

|  | **TDP-43** | | | **CAA** | | | **Atherosclerosis** | | | **Arteriolosclerosis** | | |
| --- | --- | --- | --- | --- | --- | --- | --- | --- | --- | --- | --- | --- |
| **gene** | **β** | **SE** | **P** | **β** | **SE** | **P** | **β** | **SE** | **P** | **β** | **SE** | **P** |
| *PGF* | 0.0264 | 0.0544 | 0.6277 | 0.0690 | 0.0504 | 0.1707 | 0.0551 | 0.0513 | 0.2829 | 0.0722 | 0.0484 | 0.1355 |
| *VEGFB* | 0.0059 | 0.0035 | 0.0892 | 0.0022 | 0.0031 | 0.4732 | 0.0064 | 0.0031 | **0.0387** | 0.0075 | 0.0030 | **0.0135** |
| *VEGFD* | 0.0268 | 0.2793 | 0.9235 | 0.3406 | 0.2513 | 0.1752 | -0.4942 | 0.2584 | 0.0558 | 0.1322 | 0.2455 | 0.5903 |
| *KDR* | -0.0297 | 0.2282 | 0.8963 | -0.0396 | 0.2005 | 0.8434 | -0.6964 | 0.2057 | **0.0007** | -0.3285 | 0.1972 | 0.0957 |
| *FLT4* | 0.2935 | 0.1560 | 0.0599 | 0.0925 | 0.1378 | 0.5023 | 0.1440 | 0.1431 | 0.3146 | 0.2011 | 0.1365 | 0.1407 |
| *FLT1* | 0.0197 | 0.0239 | 0.4107 | 0.0243 | 0.0217 | 0.2614 | -0.0287 | 0.0223 | 0.1969 | 0.0072 | 0.0213 | 0.7341 |
| *VEGFC* | -0.1167 | 0.3311 | 0.7246 | -0.0318 | 0.2921 | 0.9132 | -0.7296 | 0.3022 | **0.0158** | -0.5040 | 0.2942 | 0.0867 |
| *VEGFA* | -0.0016 | 0.0091 | 0.8637 | 0.0173 | 0.0083 | **0.0379** | 0.0058 | 0.0083 | 0.4862 | 0.0035 | 0.0080 | 0.6633 |
| *NRP1* | 0.0400 | 0.0722 | 0.5794 | -0.0483 | 0.0645 | 0.4540 | -0.2378 | 0.0677 | **0.0004** | -0.0899 | 0.0623 | 0.1490 |
| *NRP2* | -0.1699 | 0.1389 | 0.2214 | -0.0454 | 0.1239 | 0.7142 | -0.1839 | 0.1203 | 0.1263 | 0.1184 | 0.1179 | 0.3152 |

*Part 2*

|  | **Hippocampal Sclerosis** | | | **Gross infarcts** | | | **Microinfarcts** | | |
| --- | --- | --- | --- | --- | --- | --- | --- | --- | --- |
| **gene** | **β** | **SE** | **P** | **β** | **SE** | **P** | **β** | **SE** | **P** |
| *PGF* | 0.0875 | 0.0991 | 0.3773 | -0.0012 | 0.0567 | 0.9825 | 0.1017 | 0.0572 | 0.0754 |
| *VEGFB* | 0.0043 | 0.0065 | 0.5036 | 0.0056 | 0.0035 | 0.1050 | 0.0051 | 0.0035 | 0.1479 |
| *VEGFD* | -1.5539 | 0.6358 | **0.0145** | 0.4134 | 0.2782 | 0.1374 | 0.6194 | 0.2859 | **0.0302** |
| *KDR* | 0.2400 | 0.4193 | 0.5671 | -0.2494 | 0.2272 | 0.2724 | -0.3717 | 0.2394 | 0.1206 |
| *FLT4* | 0.1040 | 0.3012 | 0.7298 | 0.0247 | 0.1550 | 0.8735 | 0.1762 | 0.1582 | 0.2655 |
| *FLT1* | 0.0104 | 0.0459 | 0.8215 | -0.0081 | 0.0242 | 0.7374 | 0.0009 | 0.0249 | 0.9727 |
| *VEGFC* | -0.1922 | 0.6355 | 0.7623 | 0.5566 | 0.3293 | 0.0909 | 0.2130 | 0.3355 | 0.5255 |
| *VEGFA* | 0.0135 | 0.0163 | 0.4065 | -0.0073 | 0.0093 | 0.4316 | 0.0077 | 0.0093 | 0.4082 |
| *NRP1* | -0.0547 | 0.1513 | 0.7179 | -0.1324 | 0.0734 | 0.0712 | -0.0636 | 0.0753 | 0.3984 |
| *NRP2* | -0.0849 | 0.2692 | 0.7525 | -0.0967 | 0.1332 | 0.4677 | 0.0800 | 0.1359 | 0.5561 |

# Supplementary Table 11: VEGF gene associations with count and volume of gross infarcts

Note: Rows with red text are the results for genes which had significant associations with cognition.

|  | Infarct count | | | Infarct volume | | |
| --- | --- | --- | --- | --- | --- | --- |
| gene | B | SE | Pval | B | SE | Pval |
| *VEGFC* | 0.872 | 0.148 | **3.93E-09** | 1.936 | 0.555 | **0.001** |
| *VEGFB* | 0.005 | 0.002 | **0.005** | 0.017 | 0.006 | **0.005** |
| *FLT1* | 0.042 | 0.011 | **1.45E-04** | 0.087 | 0.041 | **0.035** |
| *FLT4* | 0.164 | 0.077 | **0.033** | 0.378 | 0.262 | 0.151 |
| *NRP1* | -0.036 | 0.038 | 0.346 | -0.142 | 0.124 | 0.250 |
| *PGF* | 0.052 | 0.027 | 0.057 | 0.105 | 0.096 | 0.275 |
| *NRP2* | 0.137 | 0.066 | **0.038** | 0.217 | 0.227 | 0.341 |
| *VEGFD* | -0.134 | 0.147 | 0.363 | 0.370 | 0.476 | 0.437 |
| *VEGFA* | 0.010 | 0.004 | **0.016** | 0.011 | 0.016 | 0.489 |
| *KDR* | 0.053 | 0.115 | 0.646 | 0.060 | 0.384 | 0.877 |

# Supplementary Table 12: VEGF isoform associations with pathology

Note: Used square root of continuous variables (amyloid, tangles, nft, neuritic plaques)

## Part 1: AD pathology

|  |  | **Amyloid** | | | **Tangles** | | | **NFT** | | | **Neuritic Plaques** | | |
| --- | --- | --- | --- | --- | --- | --- | --- | --- | --- | --- | --- | --- | --- |
| **isoform** | **protein-coding** | **β** | **SE** | **P** | **β** | **SE** | **P** | **β** | **SE** | **P** | **β** | **SE** | **P** |
| *FLT4-207* | n | 1.603 | 0.370 | **1.72E-05** | 1.494 | 0.403 | **2.34E-04** | 0.312 | 0.122 | **0.011** | 0.505 | 0.168 | **0.003** |
| *FLT1-201* | y | 0.064 | 0.016 | **6.03E-05** | 0.029 | 0.018 | 0.100 | 0.008 | 0.005 | 0.152 | 0.014 | 0.007 | 0.051 |
| *PGF-205* | n | 1.303 | 0.509 | **0.011** | 1.726 | 0.551 | **0.002** | 0.219 | 0.168 | 0.192 | 0.672 | 0.229 | **0.004** |
| *PGF-207* | n | 1.720 | 0.726 | **0.018** | 0.962 | 0.789 | 0.224 | 0.326 | 0.239 | 0.173 | 0.552 | 0.328 | 0.093 |
| *FLT4-201* | y | 0.324 | 0.138 | **0.019** | 0.417 | 0.150 | **0.005** | 0.080 | 0.045 | 0.078 | 0.157 | 0.062 | **0.012** |
| *VEGFB-204* | n | 0.004 | 0.002 | **0.048** | 0.006 | 0.002 | **0.010** | 0.001 | 0.001 | 0.378 | 0.002 | 0.001 | **0.023** |
| *PGF-201* | y | 0.412 | 0.213 | 0.054 | 0.739 | 0.232 | **0.001** | 0.188 | 0.070 | **0.008** | 0.138 | 0.097 | 0.153 |
| *PGF-203* | y | 0.072 | 0.041 | 0.079 | 0.140 | 0.044 | **0.002** | 0.025 | 0.013 | 0.062 | 0.046 | 0.018 | **0.013** |
| *PGF-206* | y | 0.111 | 0.086 | 0.198 | 0.135 | 0.094 | 0.153 | 0.035 | 0.028 | 0.212 | 0.070 | 0.039 | 0.071 |
| *VEGFB-202* | y | 0.012 | 0.009 | 0.215 | 0.030 | 0.010 | **0.003** | 0.006 | 0.003 | **0.039** | 0.009 | 0.004 | **0.039** |
| *FLT4-202* | y | 0.906 | 0.840 | 0.281 | 1.443 | 0.912 | 0.114 | 0.251 | 0.275 | 0.362 | 0.507 | 0.378 | 0.180 |
| *FLT1-204* | y | 0.347 | 0.345 | 0.315 | 0.739 | 0.374 | **0.049** | 0.186 | 0.114 | 0.102 | 0.192 | 0.156 | 0.220 |
| *FLT4-208* | n | 0.274 | 0.351 | 0.436 | 0.628 | 0.380 | 0.100 | 0.137 | 0.115 | 0.233 | 0.182 | 0.158 | 0.251 |
| *FLT4-211* | n | 0.267 | 0.441 | 0.546 | -0.254 | 0.479 | 0.596 | -0.176 | 0.144 | 0.222 | -0.091 | 0.199 | 0.648 |
| *FLT4-210* | n | 0.692 | 1.155 | 0.549 | 0.497 | 1.255 | 0.692 | 0.307 | 0.379 | 0.419 | 0.262 | 0.521 | 0.615 |
| *FLT4-203* | n | -1.829 | 3.204 | 0.568 | -4.995 | 3.464 | 0.150 | -1.364 | 1.039 | 0.190 | 0.004 | 1.432 | 0.998 |
| *FLT4-205* | n | 0.066 | 0.311 | 0.832 | 0.087 | 0.341 | 0.798 | 0.054 | 0.103 | 0.602 | 0.015 | 0.141 | 0.916 |
| *FLT1-202* | y | -0.004 | 0.027 | 0.874 | -0.024 | 0.030 | 0.428 | -0.001 | 0.009 | 0.869 | -0.002 | 0.012 | 0.868 |
| *VEGFB-201* | y | 0.000 | 0.004 | 0.956 | 0.009 | 0.004 | **0.033** | 0.001 | 0.001 | 0.395 | 0.003 | 0.002 | 0.134 |

## Part 2a: non-AD pathology

|  |  | **Cerebral Amyloid Angiopathy** | | | | **Cerebral Atherosclerosis** | | | | **Arteriolosclerosis** | | | |
| --- | --- | --- | --- | --- | --- | --- | --- | --- | --- | --- | --- | --- | --- |
| **isoform** | **protein-coding** | **β** | **SE** | **T** | **P** | **β** | **SE** | **T** | **P** | **β** | **SE** | **T** | **P** |
| *FLT4-207* | n | 0.736 | 0.629 | 1.169 | 0.242 | 0.118 | 0.633 | 0.187 | 0.852 | 0.611 | 0.611 | 1.001 | 0.317 |
| *FLT1-201* | y | 0.021 | 0.027 | 0.790 | 0.430 | -0.056 | 0.028 | -2.009 | **0.044** | -0.007 | 0.026 | -0.276 | 0.782 |
| *PGF-205* | n | 1.082 | 0.854 | 1.268 | 0.205 | 0.266 | 0.826 | 0.322 | 0.747 | 0.891 | 0.800 | 1.114 | 0.265 |
| *PGF-207* | n | 0.859 | 1.179 | 0.729 | 0.466 | 1.229 | 1.298 | 0.947 | 0.344 | 1.419 | 1.177 | 1.206 | 0.228 |
| *FLT4-201* | y | 0.173 | 0.226 | 0.764 | 0.445 | 0.037 | 0.234 | 0.160 | 0.873 | 0.344 | 0.223 | 1.541 | 0.123 |
| *VEGFB-204* | n | 0.000 | 0.003 | -0.041 | 0.968 | 0.002 | 0.003 | 0.623 | 0.533 | 0.008 | 0.003 | 2.401 | **0.016** |
| *PGF-201* | y | 0.202 | 0.324 | 0.622 | 0.534 | -0.369 | 0.329 | -1.124 | 0.261 | 0.713 | 0.340 | 2.097 | **0.036** |
| *PGF-203* | y | 0.057 | 0.066 | 0.861 | 0.389 | 0.030 | 0.069 | 0.433 | 0.665 | 0.094 | 0.064 | 1.462 | 0.144 |
| *PGF-206* | y | 0.154 | 0.140 | 1.105 | 0.269 | 0.200 | 0.144 | 1.390 | 0.164 | 0.144 | 0.146 | 0.983 | 0.326 |
| *VEGFB-202* | y | 0.030 | 0.016 | 1.930 | 0.054 | 0.047 | 0.016 | 3.007 | **0.003** | 0.017 | 0.015 | 1.129 | 0.259 |
| *FLT4-202* | y | 0.275 | 1.376 | 0.199 | 0.842 | -0.115 | 1.387 | -0.083 | 0.934 | -1.817 | 1.351 | -1.345 | 0.179 |
| *FLT1-204* | y | 0.929 | 0.580 | 1.601 | 0.109 | 1.497 | 0.584 | 2.564 | **0.010** | 0.174 | 0.579 | 0.301 | 0.764 |
| *FLT4-208* | n | 0.278 | 0.566 | 0.492 | 0.623 | -0.324 | 0.567 | -0.570 | 0.569 | 0.256 | 0.584 | 0.438 | 0.661 |
| *FLT4-211* | n | 0.438 | 0.762 | 0.575 | 0.566 | 1.866 | 0.808 | 2.310 | **0.021** | -0.069 | 0.727 | -0.095 | 0.924 |
| *FLT4-210* | n | -1.940 | 1.883 | -1.030 | 0.303 | 2.046 | 1.743 | 1.173 | 0.241 | 0.745 | 1.966 | 0.379 | 0.705 |
| *FLT4-203* | n | 6.774 | 5.105 | 1.327 | 0.185 | 11.193 | 5.100 | 2.195 | **0.028** | -3.017 | 4.725 | -0.639 | 0.523 |
| *FLT4-205* | n | -0.388 | 0.515 | -0.755 | 0.450 | 0.727 | 0.532 | 1.366 | 0.172 | 0.346 | 0.501 | 0.690 | 0.490 |
| *FLT1-202* | y | 0.011 | 0.042 | 0.256 | 0.798 | 0.039 | 0.042 | 0.924 | 0.356 | 0.012 | 0.042 | 0.298 | 0.766 |
| *VEGFB-201* | y | 0.003 | 0.006 | 0.527 | 0.598 | 0.019 | 0.007 | 2.903 | **0.004** | 0.013 | 0.006 | 2.069 | **0.039** |

## Part 2b: non-AD pathology

|  |  | **TDP-43** | | | **Hippocampal Sclerosis** | | | **Gross Infarcts** | | | **Microinfarcts** | | |
| --- | --- | --- | --- | --- | --- | --- | --- | --- | --- | --- | --- | --- | --- |
| **isoform** | **protein-coding?** | **β** | **SE** | **P** | **β** | **SE** | **P** | **β** | **SE** | **P** | **β** | **SE** | **P** |
| *FLT4-207* | n | 1.324 | 0.690 | 0.055 | 1.513 | 1.271 | 0.234 | 0.054 | 0.689 | 0.937 | 0.567 | 0.705 | 0.421 |
| *FLT1-201* | y | 0.012 | 0.030 | 0.680 | 0.025 | 0.055 | 0.644 | -0.001 | 0.030 | 0.964 | 0.028 | 0.030 | 0.354 |
| *PGF-205* | n | -0.320 | 0.940 | 0.734 | 1.676 | 1.634 | 0.305 | -0.292 | 0.942 | 0.757 | 0.723 | 0.955 | 0.449 |
| *PGF-207* | n | 0.999 | 1.331 | 0.453 | 1.740 | 2.097 | 0.407 | -0.127 | 1.326 | 0.923 | -0.735 | 1.401 | 0.600 |
| *FLT4-201* | y | 0.335 | 0.256 | 0.190 | 0.436 | 0.475 | 0.359 | 0.111 | 0.255 | 0.663 | 0.418 | 0.260 | 0.109 |
| *VEGFB-204* | n | 0.007 | 0.004 | 0.081 | 0.006 | 0.006 | 0.300 | 0.003 | 0.004 | 0.472 | 0.008 | 0.004 | **0.037** |
| *PGF-201* | y | 0.246 | 0.349 | 0.479 | 0.200 | 0.754 | 0.791 | 0.097 | 0.393 | 0.806 | -0.446 | 0.475 | 0.348 |
| *PGF-203* | y | 0.040 | 0.072 | 0.578 | -0.028 | 0.143 | 0.845 | -0.011 | 0.075 | 0.883 | 0.136 | 0.076 | 0.074 |
| *PGF-206* | y | -0.111 | 0.161 | 0.493 | 0.021 | 0.320 | 0.947 | 0.209 | 0.160 | 0.191 | 0.342 | 0.162 | **0.035** |
| *VEGFB-202* | y | 0.013 | 0.017 | 0.467 | 0.040 | 0.032 | 0.207 | 0.041 | 0.017 | **0.019** | 0.014 | 0.018 | 0.426 |
| *FLT4-202* | y | 0.140 | 1.530 | 0.927 | 4.196 | 2.648 | 0.113 | -0.222 | 1.539 | 0.885 | 0.778 | 1.573 | 0.621 |
| *FLT1-204* | y | 1.122 | 0.621 | 0.071 | 1.494 | 1.074 | 0.164 | 0.818 | 0.635 | 0.197 | 0.219 | 0.651 | 0.736 |
| *FLT4-208* | n | 0.196 | 0.652 | 0.764 | -0.267 | 1.249 | 0.831 | -0.283 | 0.645 | 0.661 | 0.086 | 0.660 | 0.896 |
| *FLT4-211* | n | 2.051 | 0.808 | **0.011** | -1.845 | 1.944 | 0.343 | 0.302 | 0.805 | 0.708 | -1.058 | 0.886 | 0.232 |
| *FLT4-210* | n | -2.619 | 2.434 | 0.282 | 1.462 | 3.694 | 0.692 | 2.964 | 2.208 | 0.179 | 4.464 | 2.262 | **0.048** |
| *FLT4-203* | n | -5.676 | 5.601 | 0.311 | -51.074 | 41.648 | 0.220 | -3.311 | 5.823 | 0.570 | 5.751 | 5.810 | 0.322 |
| *FLT4-205* | n | -0.069 | 0.568 | 0.904 | 0.014 | 1.069 | 0.990 | 0.059 | 0.572 | 0.919 | 0.477 | 0.581 | 0.412 |
| *FLT1-202* | y | 0.007 | 0.041 | 0.874 | -0.027 | 0.096 | 0.781 | -0.115 | 0.068 | 0.093 | -0.089 | 0.073 | 0.222 |
| *VEGFB-201* | y | 0.007 | 0.007 | 0.295 | 0.002 | 0.014 | 0.907 | 0.011 | 0.007 | 0.141 | 0.011 | 0.007 | 0.143 |
